# A comparative study of Chinese medicine quality of life assessment scale (CQ-11D) and EQ-5D-5L and SF-6D scales based on Chinese population

**DOI:** 10.1007/s11136-023-03512-z

**Published:** 2023-09-11

**Authors:** Jiameng Zhou, Longchen Xu, Jie Pan, Muqing Wang, Pingda Zhou, Wei Wang, Siqi Lu, Wentao Zhu

**Affiliations:** School of Management, Beijing University of Chinese Medicine, Beijing, 100029 China

**Keywords:** Health-related quality of life, Health utility, CQ-11D, EQ-5D-5L, SF-6D

## Abstract

**Purpose:**

To measure health-related quality of life in the Chinese population using three universal health utility scales (CQ-11D, EQ-5D-5L, and SF-6D) and to compare the differences in the results obtained by the different scales to provide a reference for future utility on health-related quality of life in the Chinese population.

**Methods:**

According to the Chinese population's distribution area, gender, and age, quota sampling was conducted. Three scales, CQ-11D, EQ-5D-5L, and SF-6D, whose results were self-reported, were collected in succession after collecting respondents' demographic information. The health utility value and floor/ceiling effect were explained. Bland–Altman was used to evaluate the consistency, the intraclass correlation coefficient was used to evaluate the correlation, and the receiver operating characteristic curve was used to evaluate the discriminative validity of the scale.

**Results:**

The mean utility values of the CQ-11D, EQ-5D-5L, and SF-6D scales, respectively, were 0.891, 0.927, and 0.841. The floor effect did not appear in any of the three scales, but the ceiling effect did, and the EQ-5D-5L ceiling effect was the most severe. The limits of the agreement interval for CQ-11D versus EQ-5D-5L in the total sample population were (-0.245,0.172); for CQ-11D versus SF-6D, they were (− 0.256,0.354); and for EQ-5D-5L versus SF-6D, they were (-0.199,0.371). The consistency of the three scales is satisfactory overall. In the total population, the intraclass correlation coefficient between CQ-11D and EQ-5D-5L was 0.709, while EQ-5D-5L and SF-6D were 0.0.565 and that between EQ-5D-5L and SF-6D was 0.472. According to the subject operation curve results, the area under the curve for the total sample population of CQ-11D was 0.746, EQ-5D-5L was 0.669, and SF-6D was 0.734.

**Conclusion:**

The CQ-11D is inferior to the EQ-5D-5L, but superior to the SF-6D. There is a strong correlation between the health utility values of the total population as measured by the three scales and those of the healthy population. The CQ-11D scale is the most sensitive to differences between populations and diseases.

**Supplementary Information:**

The online version contains supplementary material available at 10.1007/s11136-023-03512-z.

Health-related quality of life (HRQoL) is a subjective concept that reflects the quality of life and measures people's physical, psychological, social, and spiritual personal role functions [1]. As the world's most widely utilized universal health utility scales, the EQ-5D and SF-6D can calculate quality-adjusted life years (QALYs). Cost-utility analysis (CUA) provides evidence-based support for the economic evaluation of health interventions.

Relevant studies have demonstrated the existence of a ceiling or floor effect in the measurement of HRQoL for scales EQ-5D-5L and SF-6D [2–4]. However, the ceiling effect of scale EQ-5D-5L has been reduced relative to scale EQ-5D-3L; nevertheless, it still exists [5]. In addition, these scales may need to accurately reflect the health preferences and characteristics of the Chinese population in terms of selection, emphasis, and description of health status due to research and development based on foreign populations. In recent years, the scale health status description system has placed a greater emphasis on the participation of the general population in the construction process, and the results based on measurements of the general population have become more extrapolated and universal [6].

In addition, due to the differences in dimensions and levels of different scales, there may be differences in measurement results when applied to different populations; therefore, it is crucial to conduct head-to-head comparisons of different utility measurement instruments.

In empirical studies, EQ-5D and SF-6D are predominantly used to measure HRQoL, and there needs to be more research based on Chinese population characteristics or the perspective of traditional Chinese medicine. Consequently, this study utilizes the utility-scale evaluation scale of quality of life in Chinese medicine (CQ-11D), which is based on the research and development of the Chinese population, the theory of traditional Chinese medicine, and the health concept of traditional Chinese medicine, as well as the globally prevalent EQ-5D-5L and SF-6D [7]. The study will further compare the distribution, correlation and consistency of the three scales on the health utility measurement results of the general population in China, and conduct analysis in combination with the influencing factors, aiming to explore the differences in the measurement results of the three scales, provide an empirical basis for the comparative study of utility scales developed based on the Chinese population and provide reference for the improvement of HRQOL in Chinese general population and the selection of appropriate quality of life assessment tools for researchers.

## Data and methods

### Research objects

This study covers the period from January 2022 to December 2022 and is based on research conducted by Chinese citizens across the nation. Through the seven investigated geographic divisions, each partition of 2 ~ 6 selected representatives of provinces, autonomous regions, and municipalities directly under the central government, based on prior research experience and the area, gender, and age distribution of quota sampling of the general Chinese population [8]. The local area of the investigator is chosen for the interview survey. The researcher conducts research through a one-on-one or face-to-face questionnaire survey and searches for interviewees through encounter sampling in the public area (e.g., street, community, school) within the jurisdiction of the local area [9, 10]. The inclusion criteria were as follows: (1) participants must be at least 16 years old; (2) they must be Chinese citizens with Chinese nationality; (3) they must have resided in mainland China for the past five years; (4) they must have comprehended the research's background information and agreed to participate. The exclusion criteria for respondents were as follows: (1) had difficulties in listening, speaking, reading, or writing or could not comprehend the survey content; and (2) had a mental disorder.

All investigations were conducted with the informed consent of the subjects and with Ethics Committee approval. The survey was conducted with questionnaires, and investigators questioned interviewees with appropriate training. The basis for determining the group of chronic patients is that the respondents report having chronic diseases confirmed by doctors. In addition, the respondents' sociodemographic characteristics (age, gender, income, smoking, drinking, and physical activity) were collected, and their health status was subsequently determined using the EQ-5D-5L, SF-6D, and CQ-11D scales.

### Scales

#### Background and purpose of developing the CQ-11D scale

Related studies have shown that EQ-5D-5L scales and SF-6D scales have ceiling or floor effect in measuring HRQOL [11–13]. More importantly, although these scales have good reliability and validity and are widely used, they are based on scales developed by foreign population. The selection, focus and description of health status may not accurately reflect the health preferences and characteristics of the Chinese population [6], especially the international universal quality of life scale can not fully reflect the characteristics of health output of traditional Chinese medicine intervention. The health output of traditional Chinese medicine intervention is insufficient or even underestimated. From the point of view of quality of life and patients, CQ-11D aims to develop a quality of life scale based on Chinese population, which can objectively evaluate the reported outcome of patients with traditional Chinese medicine intervention. By following the development procedure of the international scale, based on the relevant concepts of the quality of life of the World Health Organization (WHO), according to the basic contents of the quality of life, referring to the relevant contents of the foreign universal scale, on the basis of the theory and concept of health of traditional Chinese medicine, combined with the characteristics of traditional Chinese medicine intervention and Chinese culture, consult experts in the field of traditional Chinese medicine, scale and quality of life. Construct the theoretical framework of the quality of life evaluation scale based on the theory of traditional Chinese medicine, and then define the nature and applicable population of the scale according to the purpose of the development of the scale. based on the patient report outcome, a quality of life evaluation scale based on traditional Chinese medicine theory was developed for the evaluation of health quality of life of people who received intervention of traditional Chinese medicine.

#### Construction of CQ-11D scale health utility score system

The health utility score system of the Chinese medicine Quality of life-11Dimensions (CQ-11D) is based on the health preference of the Chinese population, which is constructed by using the discrete choice experiment (DCETTO) with survival time, and used in conjunction with the corresponding TCM quality of life scale to calculate the subjects' health utility value. The study was designed to recruit at least 2400 respondents across mainland China to complete one-to-one, face-to-face questionnaire surveys. A total of 2,586 people were invited to participate in the survey and 2498 valid questionnaires were completed (a completion rate of 96.60%). The conditional logit model was ultimately selected to construct a health utility scoring system for CQ-11D utility measurement. The measurable health utility value range was − 0.868 ~ 1 [7].

The utility values integral system Zhu Wentao and Luo N created for the Chinese population was adopted for the CQ-11D and EQ-5D-5L scales, respectively. However, because the utility point system based on the Chinese population still needed to be developed, the utility value integral system based on the Hong Kong population was adopted for scale SF-6D [10, 14, 15].

#### Dimensional comparison of CQ-11D, EQ-5D-5L and SF-6D

CQ-11D contains 11 items: movement and self-care, appetite, stool, quality of sleep, spirit (being alive, energetic, and focused), dizziness (feeling dizzy in the mind, with eyes closed for minor cases, or spinning in front of the scene in serious cases, inability to stand), palpitations (feeling restless), pain, fatigue, irritability, anxiety (worried, anxious, nervous, restless), and depression (frustrated, lack of interest in doing things, no fun, low energy). Mobility, self-care, daily activities, pain and discomfort, and anxiety or depression are the five dimensions of the EQ-5D-5L. Each dimension has five levels: no problems, slight problems, moderate problems, severe problems, and extreme problems [16]. SF-6D comprises 6 dimensions: physical function, role limitation, social function, pain, mental health, and vitality, each dimension has four to six levels.

The three scales differed in the number of dimensions and measured health status. The CQ-11D scale has 11 entries, each entry has 4 levels, and can measure 4^11^ (ie 4,194,304) health states. The EQ-5D-5L scale has 5 dimensions, each with 5 levels, and can measure 5^5^ (ie 3,125) health states. The SF-6D scale has 6 dimensions, each with 4 to 6 levels, and can measure 18,000 health states. The three scales have certain similarities in terms of movement, pain, and mental health, but the items of CQ-11D, such as energy and energy, bowel movements, sleep quality, and appetite, are not included in the other two scales.

### Quality control

After the investigation has been completed, the survey members of the research group must review the data and eliminate the data obtained if the investigator fails to follow the Investigation Manual. This can ensure the investigation's quality.

### Data analysis

Four distinct analyses were performed on the collected data: descriptive analysis, health utility distribution and ceiling/floor effect, consistency and correlation analysis, and a scale sensitivity study. Since the health utility measurement obtained through the three-scale utility value integral system can be used directly for quantitative comparison of measurement results, the health utility value was chosen as the primary analysis index in this study.

In the descriptive analysis, frequency (proportion) was used to describe the categorical variables, and the histogram was used to observe the distribution characteristics of the health utility values across the three scales. In the analysis of ceiling and floor effects, it is generally accepted that more than 15 percent of the dimension or total score reaching the highest or lowest score will be considered to have a ceiling or floor effect on the dimension or total score [17]. The intraclass correlation coefficient and the Bland–Altman method were used to examine correlation and consistency, and the study index was the health utility value of the three scales. Utilizing receiver operating characteristic (ROC) curves, the ability to distinguish the four subgroups specified by the VAS based on varying scores on different scales was demonstrated (0–65, 66–85, 86–95, and 96–100) [18]. The ROC curve provides information regarding the scale score's sensitivity and specificity (health utility). The area under the curve (AUC) was measured between 0.5 (undifferentiated accuracy) and 1.0 (perfect accuracy). The greater the value of AUC, the higher the differentiation accuracy [19].

### Statistical methods

P < 0.05 was considered statistically significant. SAS 9.2 was used for descriptive analysis, SPSS 26 was used to draw the subject working curve, R language software was used to calculate the intra-group correlation coefficient, and histograms and a Bland–Altman chart were produced.

## Results

### Sample research quota situation and distribution of research cities

The survey was conducted according to seven geographical regions in China, with 3–7 representative provinces, autonomous regions and municipalities directly under the Central Government selected in each region. Based on the existing research experience and according to the distribution area, gender and age distribution of the general population in China, the quota sampling was carried out [8, 20], The survey area covers all major cities in seven sub-regions of China, covering seven geographical divisions of North China, Northeast China, East China, Central China, South China, Southwest China and Northwest China, totaling 37 provinces, cities, autonomous regions, municipalities directly under the Central Government and special administrative regions. The area where the investigator was located was selected for interview survey. Investigators looked for interviewees in the public areas (streets, communities, schools, etc.) within the jurisdiction of the area where they are located. The general representative population in China was investigated in the form of one-to-one and face-to-face questionnaire, as shown in Table 1 below. The survey was carried out from February to November 2022, including three different survey parts. 5000 questionnaires were allocated according to age and gender, During the research period, the team recruited a total of 196investigators, interviewed a total of 5018 respondents, and the number of effective interviews was 5000, as shown in Table 2 below.

**Table 1:** Survey of cities in sample quota areas

Geographical divisions	Sample	Representative provinces, municipalities, autonomous regions and municipalities directly under the Central Government
North	500	Beijing
		Tianjin
		Shanxi
		Hebei
Northeast	380	Heilongjiang
		Jilin
		Liaoning
East	805	Shanghai
		Jiangsu
		Zhejiang
		Anhui
		Jiangxi
		Shandong
		Fujian
Central	1528	Henan
		Hubei
South	672	Guangdong
		Guangxi
		Hainan
		Hong Kong
		Macao
Southwest	740	Chongqing
		Sichuan
		Guizhou
		Yunnan
		Tibet
Northwest	375	Shaanxi
		Gansu
		Qinghai
		Ningxia
		Xinjiang
		Inner Mongolia

**Table 2:** Sample survey quota

Age	Gender	
	Male (person)	Female (person)
15–24	300	300
25–34	500	500
35–44	400	400
45–54	500	500
55–64	400	400
65–74	200	200
≥ 75	200	200
Total	2500	2500

The research quota was based on the relevant data of China Statistical Yearbook 2021

### Sociodemographic characteristics

This study examined 5000 members of the general population, including 2281 males and 2719 females ranging in age from 16 to 80. The specific distribution is shown in Table 3; the means (median) of the utility of scales CQ-11D, EQ-5D-5L, and SF-6D are 0.891 (0.940), 0.927 (0.951), and 0.841 (0.888), respectively.

**Table 3:** Sociodemographic characteristics of the research sample (*N* = 5000)

Social demographic characteristics	Research sample (*n*)	Ratio (%)
Sex		
Men	2286	45.72
Women	2714	54.28
Area		
North China	500	10.00
Northeast of China	380	7.60
Central China	1528	30.56
East China	805	16.10
Southern part of China	672	13.44
Southwest of China	740	14.80
Northwest of China	375	7.50
Age		
16–24 years old	1262	25.24
25–34 years old	788	15.76
35–44 years old	699	13.98
45–54 years old	964	19.28
55–64 years old	573	11.46
65–74 years old	314	6.28
75 years and older	400	8.00
Current family and marital status		
Married and childbearing	2872	57.44
Married with no children	177	3.54
Unmarried	1809	36.18
Divorce/widowhood	135	2.70
Else	7	0.14
Nation		
The Han nationality	4436	88.72
Minority	564	11.28
Occupation		
Incumbency	2288	45.76
Retire	621	12.42
Student	1411	28.22
Unemployment	494	9.88
Else	186	3.72
Census register		
Village	2152	43.04
City	2848	56.96
Degree of education		
Primary and below	454	9.08
Junior high school	780	15.60
Senior high school/technical secondary school	1434	28.68
Undergraduate college	1989	39.78
Master degree or above	343	6.86
Medical insurance type		
Basic medical insurance for urban workers	1807	36.14
Basic medical insurance for urban residents	1387	27.74
New rural cooperative medical insurance	1579	31.58
Medical insurance for retired cadres	91	1.82
Else	136	2.72
Whether they have received or are receiving Chinese medicine treatment		
Yes	2028	40.56
No	2972	59.44
Whether you have a chronic disease		
Yes	2286	46.72
No	2714	54.28
Smoke		
Never	3761	75.22
Occasionally (1–2 days a week)	365	7.30
Often (3–6 days per week)	249	4.98
Smoking almost every day	411	8.22
Quit smoking	214	4.28
Drinking		
Never	2848	56.96
Occasionally (1–2 days a week)	1446	28.92
Often (3–6 days per week)	273	5.46
Drinking almost every day	121	2.42
Alcohol abstinence	312	6.24
Exercise situation		
Exercise every day	1096	21.92
3–6 times a week	852	17.04
1–2 times a week	1486	29.72
Barely exercise	1316	26.32
Not quite clear	25	5.00
Average monthly earnings		
0–1300 rmb	905	18.10
1301–3300 rmb	1612	32.24
3301–6300 rmb	1389	27.78
6301–13,000 rmb	763	15.26
13,001–21,000 rmb	158	3.16
21,001–42,000 rmb	48	0.96
More than 42,001 rmb	125	2.50
Changes in health status over the past year		
No change	2009	40.18
Got better	1279	25.58
Become bad	896	17.92
Not quite clear	816	16.32

### Distribution of measurements and ceiling/floor effects

**Fig. 1:**
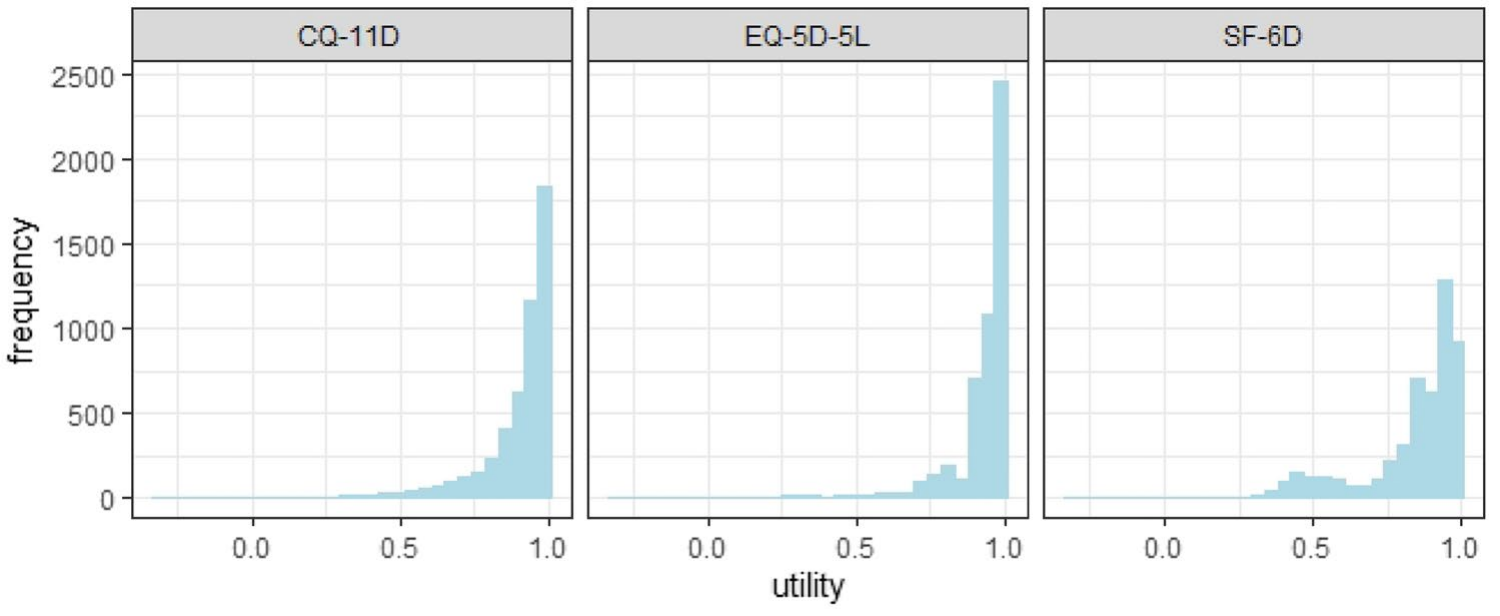
Distribution of health utility values of the total samples of the three scales

Figure 1 demonstrates that none of the three measurement techniques conform to the normal distribution and that the overall utility value is high. The measured utility values for CQ-11D ranged from -0.301 to 1. The histogram revealed that data continuity was satisfactory and that both medium and high utility values were represented. In the area of low efficiency, only a tiny quantity of fault data existed. The distribution of EQ-5D-5L utility value was relatively concentrated, ranging from -0.201 to 1, indicating a significant ceiling effect (49.04%). The distribution range of SF-6D is 0.036–1.

The ceiling effect was observed in all three scales, with EQ-5D-5L exhibiting the highest level (19.42%), SF-6D exhibiting the lowest level (18.30%), and CQ-11D falling in between (18.64%). The floor effect was not observed on each of the three scales. The average health utility value of the healthy population is greater than that of the sick population, and the distribution results of the health utility value of the other groups are comparable to the overall sample results, which are not repeated here.

### Consistency and correlation test of the measurement results

In this study, a Bland–Altman comparison was performed on the utility values of the three scales. Under the assumption of sampling error, the confidence intervals of the limits of agreement (Limits of Agreement, LoA) between CQ-11D and SF-6D for the five groups are wider than those for the other two groups.

In the total population, more than 94.34% of samples from groups CQ-11D vs. EQ-5D-5L and CQ-11D vs. SF-6D were within the LoA confidence interval. In contrast, only 91.46% of samples from group EQ-5D-5L vs. SF-6D were found. It indicates that the measurement value of EQ-5D-5L was greater than that of SF-6D (427 samples) Tables 4, 5 and 6.

**Table 4:** Bland–Altman analysis results

Scale	LoA CI	Range Number of samples	Outside the range Number of samples (%)
CQ-11D VS EQ-5D-5L	(− 0.245,0.172)	4717	94.34
CQ-11D VS SF-6D	(− 0.256,0.354)	4584	91.68
EQ-5D-5L VS SF-6D	(− 0.199,0.371)	4573	91.46

**Table 5:** ICC analysis results

Scale	ICC	P
CQ-11D VS EQ-5D-5L	0.709	< 0.001
CQ-11D VS SF-6D	0.565	< 0.001
EQ-5D-5L VS SF-6D	0.472	< 0.001

**Table 6:** ROC results

The crowd	Scale	AUC	STD	P
Entire population	CQ-11D	0.746	0.009	0.000
	EQ-5D-5L	0.669	0.010	0.000
	SF-6D	0.734	0.010	0.000
Healthy population	CQ-11D	0.710	0.012	0.000
	EQ-5D-5L	0.610	0.013	0.000
	SF-6D	0.702	0.012	0.000
Chronic diseases population	CQ-11D	0.755	0.017	0.000
	EQ-5D-5L	0.704	0.017	0.000
	SF-6D	0.735	0.018	0.000
A single chronic disease	CQ-11D	0.721	0.022	0.000
	EQ-5D-5L	0.676	0.021	0.000
	SF-6D	0.720	0.023	0.000
Multiple chronic diseases	CQ-11D	0.798	0.026	0.000
	EQ-5D-5L	0.780	0.027	0.000
	SF-6D	0.736	0.029	0.000
Hypertension	CQ-11D	0.743	0.035	0.000
	EQ-5D-5L	0.701	0.035	0.000
	SF-6D	0.707	0.040	0.000
Fatty liver	CQ-11D	0.799	0.049	0.000
	EQ-5D-5L	0.717	0.052	0.000
	SF-6D	0.770	0.059	0.000
Chronic gastritis	CQ-11D	0.792	0.043	0.000
	EQ-5D-5L	0.651	0.006	0.015
	SF-6D	0.687	0.070	0.007

Bartko first proposed the intraclass correlation coefficient (ICC) in 1966; it can be used to evaluate the consistency or reliability of different quantitative measurement methods [22]. In this study, the utility values of the three scales were compared in pairs to determine their ICC. All P values were statistically significant (< 0.05) and positively correlated. From the scale, the results of all populations demonstrated the following ICC: CQ-11D VS EQ-5D-5L > CQ-11D VS SF-6D > EQ-5D-5L VS SF-6D. The correlation between CQ-11D and scale EQ-5D-5L was high, whereas the correlation between scale EQ-5D-5L and scale SF-6D was low.

### ROC analysis results

**Fig. 2.**
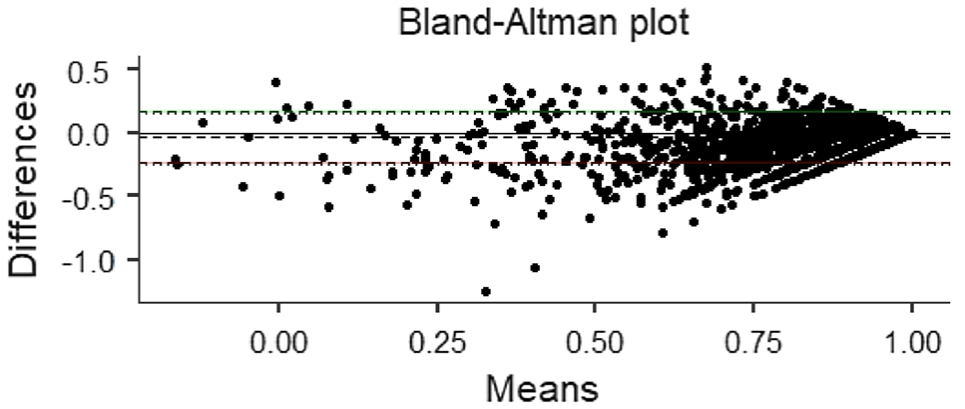
Consistency of comparison between population samples CQ-11D and EQ-5D-5L Bland–Altman results

**Fig. 3.**
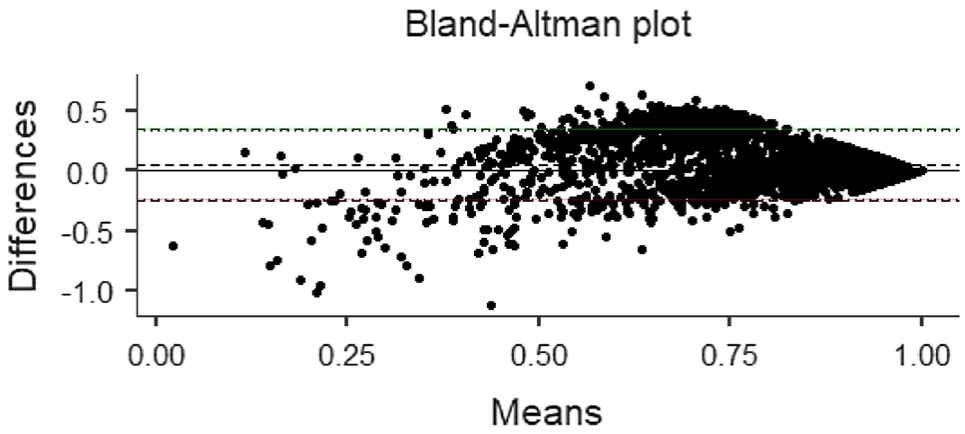
Consistency of comparison between population samples CQ-11D and SF-6D Bland–Altman results

**Fig. 4.**
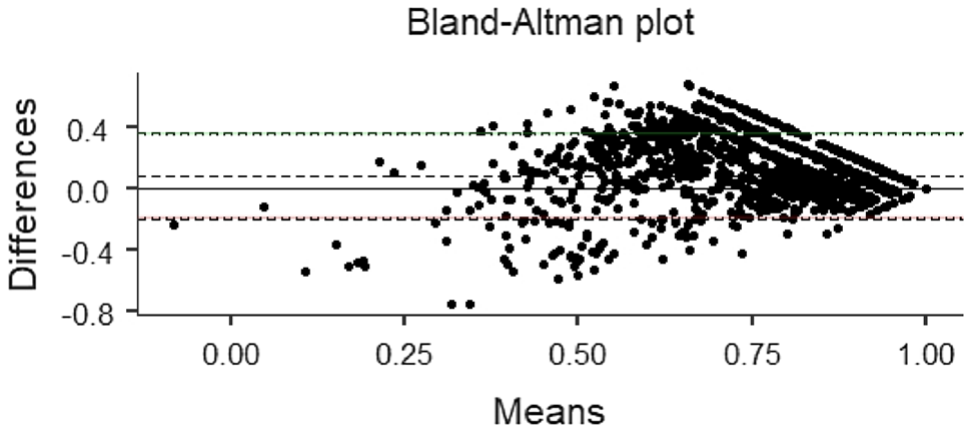
Consistency of comparison between population samples EQ-5D-5L and SF-6D Bland–Altman results

**Fig. 5.**
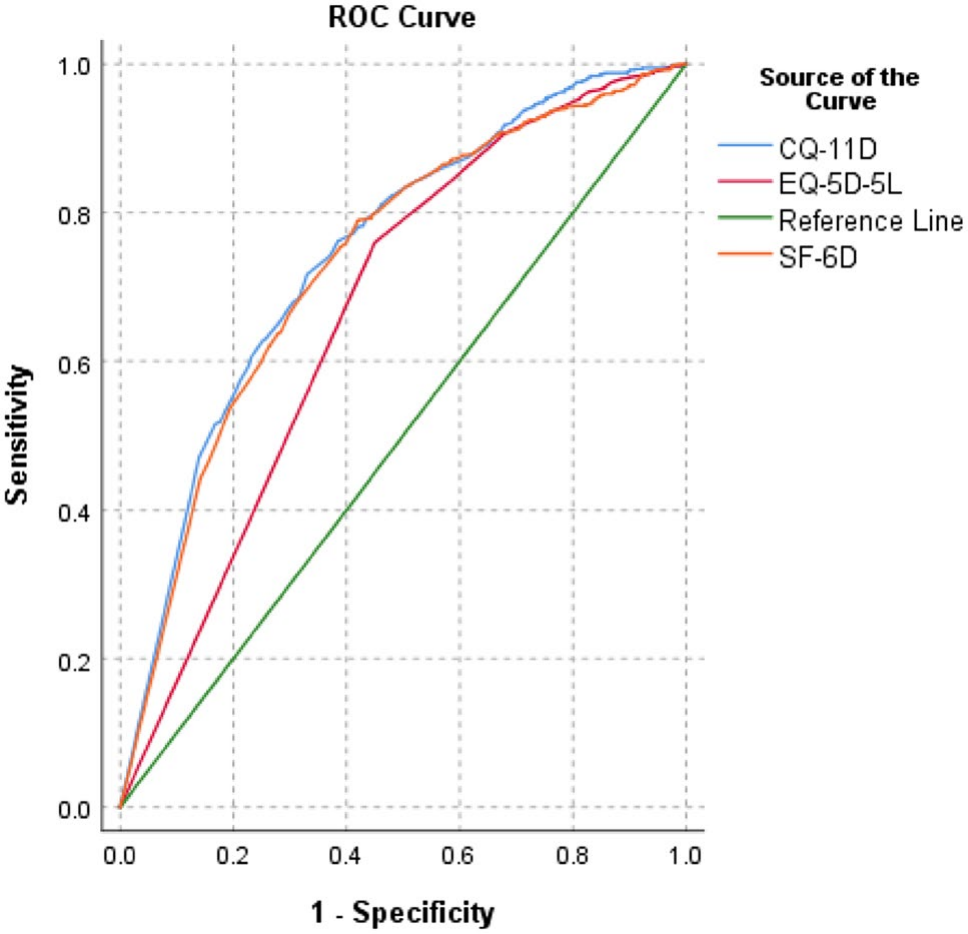
ROC curve of the entire population

The ROC analysis results show that the AUC of the health utility values of the three scales are higher than 0.5 in the general population, healthy population and chronic patient group. Combined with the model quality evaluation results, it is considered that the model quality of the three scales is high, and the results of the three scales are meaningful.In overall population, the discrimination of the CQ-11D scale measurement results (0.746) is better than that of the SF-6D scale (0.734) and the EQ-5D-5L scale (0.669); In healthy population, the discrimination of CQ-11D scale measurement results (0.710) is better than SF-6D scale (0.702) and EQ-5D-5L scale (0.610); In chronic patient group, the discrimination of the CQ-11D scale measurement results (0.755) is better than the SF-6D scale (0.735) and the EQ-5D-5L scale (0.704). In addition, the overall representativeness of the RoC of the three scales is good, but the sample size of the healthy population and chronic patient group is relatively small, which may have certain limitations on the ROC analysis results (Figs. 2, 3, 4 and 5).

## Discussion

The dimension of CQ-11D scale is the most among the three scales, and it also involves the most abundant categories, and it is also the widest in the range of health utility measurement results. The larger the measurement dimension and measurement range, the more comprehensive the results of the meter measurement to a certain extent. The main results are as follows: (1) after subdividing the health utility value for different populations, this study selects the areas where health utility values gather to observe its distribution. The results also show that the CQ-11D scale has a good measurement performance in terms of the measurement range and continuity of health utility values. (2) Compared with the EQ-5D scale, CD-11D did not show obvious ceiling effect and no floor effect. (3) in previous studies, the CQ-11D scale has been proved to have a good responsiveness [21, 22], and in this study, the responsiveness of CQ-11D is also well reflected. Although the dimensions of the CQ-11D scale are more than the other two scales, because the dimensions of the scale cover the basic concepts of traditional Chinese medicine and are close to life, it does not reflect the inadaptability in the survey. Therefore, the CQ-11D scale satisfies the richness of connotation and the convenience of filling in the scale to some extent.

This study selected representative samples of the Chinese population and developed CQ-11D to measure HRQoL in the Chinese population. CQ-11D was then combined with EQ-5D-5L and SF-6D, two international universal scales, to compare measurement results, which can provide relevant evidence for researchers and policymakers and has specific theoretical and practical implications. The results indicated that the Chinese population's overall health utility was relatively high. The health utility value measured by respondents based on scale CQ-11D was greater than that measured by scale SF-6D and less than that measured by scale EQ-5D-5L, and there were differences in the measurement results across scales.

### The health attributes of the population covered by the measurement results of CQ-11D are better

The measurement results show that CQ-11D can reflect better coverage attributes and sensitivity in the measurement of HRQoL in the Chinese population, the measurement scope of quality of life is broader, and the measurement results have certain advantages in the Chinese population. It is found that the utility distribution of Scale CQ-11D is continuous and wide, which can cover most people well and reflect the health utility of people with different health statuses.

The results of the measurements revealed that the floor effect did not appear on any of the three scales, whereas the ceiling effect appeared on all of them. SF-6D had the lowest ceiling effect, at 18.30%, close to the critical value. Moreover, EQ-5D-5L has the highest ceiling effect, nearly 50%. In contrast, the EQ-5D-5L scale is relative to the EQ-5D-3Lscale expansion in dimensions, and the empirical study demonstrates that the EQ-5D-5L ceiling effect relative to EQ-5D-3L has decreased﻿ [12]. However, the results continue to indicate a higher ceiling effect. In healthy individuals, the ceiling effect of the Scale EQ-5D-5L was greater than 50%; however, in patients with multiple chronic diseases and the worst theoretical health status, the ceiling effect of the Scale EQ-5D-5L still exceeded the critical value of 15%.

Even though there are some differences in the distribution of measurement results across different populations, they all indicate that the EQ-5D-5L scale focuses primarily on areas with high utility value. In contrast, the SF-6D scale has the narrowest distribution range. In clinical research, a value between 0 and 1 is typically employed to represent quantitative health status results. 0 represents death, while 1 represents perfect health. People who are unconscious or bedridden for an extended time, accompanied by severe pain, and afflicted with a severe tumor disease may experience an adverse health effect worse than death. A patient with multiple chronic diseases, a lengthy course of medication, and combined medication may experience adverse reactions. This individual may have a poor health status. In this study, the measurement results of CQ-11D and EQ-5D-5L are negative. However, this does not imply that the measurement performance of these two scales is necessarily better than that of SF-6D, which is primarily related to the construction method of the integral utility system attached to the scale and the construction result of the final utility integral system.

### The measurement results of the three scales have high consistency, but there are significant differences in the correlation results

The consistency of the CQ-11D and EQ-5D-5L is higher in samples from the total population, whereas the consistency of the SF-6D and other two scales is lower. It may be due to the SF-6D and other scales gap being too broad, considering the possible difference in connotation and its evaluation measurement. The EQ-5D-5L contains the primary factors influencing the quality of life with concise and well-defined dimensions. The dimensions and levels of scale SF-6D are more robust than those of scale EQ-5D-5L, which, to some extent, facilitates the incorporation of fillers into their situations. In addition, differences in the construction of the point system and the measurement process between the three scales may also contribute to the low consistency of the three measurement results.

As the health of a population improves or deteriorates, three types of scale measurements result in different changes, and the results of the three types of scale measurements vary based on the state of health. The results outside the interval suggest that either the measured value of EQ-5D-5L or SF-6D is either excessively high or inadequately low. It could be caused by the following: (1) The quantity and connotation of scale dimensions and levels (items) are vastly distinct; (2) there are significant differences in expression. EQ-5D-5L, for instance, indicates the respondents' situation "on that day," whereas SF-6D indicates the respondents' situation "during the past four weeks." In the remaining groups, the results of the three scales were consistent [7, 13, 23].

Like the consistency measurement results, the icc demonstrated a high correlation between CQ-11D and EQ-5D-5L across entire samplings. But the correlation between the measurement results of EQ-5D-5L and scale SF-6D was less than 0.5, while the consistency results showed good consistency between the two. It could be attributed to healthy individuals' relatively calm measurement state and their insensitivity to using the intra-group correlation coefficient to measure the results. In different populations, the ICC of the patients was higher than that of the healthy people in the same group.

### There are differences in the performance of the three scales in the measurement of different groups of people and types of diseases

The ROC curve is drawn in AUC as judgment indexes. The result indicates that in the total population, health, population, and sick population (including the risk of a single scale of chronic disease and multiple chronic diseases), the ability of CQ-11D to differentiate between different health crowd effects is superior. Furthermore, it implies that CQ-11D is superior to EQ-5D-5L and SF-6D in measuring sensitivity (differentiation) in the general population in China.

It may be due to several dimensions and items in CQ-11D, particularly in dimension, which is influenced by the holistic view of traditional Chinese medicine and focuses on the overall status and feelings of the participants and is somehow more sensitive to the changes in the health status of the Chinese population [7].

In measuring patients with simple obesity, CQ-11D demonstrates superior discriminative validity and greater sensitivity than the other two scales, depending on the disease being assessed. Furthermore, hypertension and chronic gastritis, results demonstrated that the CQ-11D measurement results had a larger area under the curve and a higher sensitivity than the other two scales.

When different scales are used for comparative research, the adaptation of scales to specific situations should be discussed. No single gold standard exists. Previous research has demonstrated that the Scale EQ-5D-5L is simple to comprehend and is less affected by the respondents' educational level and comprehension ability. In contrast, the Scale SF-6D performs better in the slow process of disease measurement. Therefore, it is recommended that researchers choose corresponding scales based on their research's measurement objectives and scale characteristics. Since the three scales differ significantly in dimension and level, two or more scales can be utilized in the study to reflect the health status of respondents accurately [12, 24].

## Boundedness

There is some heterogeneity in this study: (1) The sampling method used in this study is quota sampling. Quota sampling gives investigators more rights of free investigation in each category. Although the results of many quota surveys are close to the results of Stratified sampling in probability sampling, it cannot be determined whether the sample is representative enough, and the results obtained cannot be well extrapolated to the general population of China. In future research, we will try our best to obtain survey data through probability sampling. (2) Considering the large sample size and convenience of this study, the order of the three scales was not randomly set during the research process, which may have an impact on the survey data and lead to random bias. We will consider this issue in the subsequent research process and randomly set the order of the three scales. At the same time, we explained this issue in the limitations section of the article. (3) Cross-sectional data can not study the HRQoL results of different populations and individuals in China under time changes; in the sampling process, the sample size of some populations (such as the age group of 16–25 years old) is slightly more than the quota, which may have a certain impact on the study. (4) in the sampling process, the sample size of some populations (such as the age group of 16–25 years old) is slightly more than the quota, which may have a certain impact on the study.

### Supplementary Information

Below is the link to the electronic supplementary material.

Supplementary file1 (DOCX 20 kb)

## Data Availability

The data that support the findings of this study are available from the corresponding author upon reasonable request.
