# Supplementary material for: A comparative study of Chinese medicine quality of life assessment scale (CQ-11D) and EQ-5D-5L and SF-6D scales based on Chinese population
Source: Qual Life Res. 2023 Sep 11;33(1):113–22. doi: 10.1007/s11136-023-03512-z (PMC10784339; doi:10.1007/s11136-023-03512-z)
Supplement: Supplementary file 1 — Supplementary file1 (DOCX 20 kb) [file 11136_2023_3512_MOESM1_ESM.docx]

**Supplementary Appendix**

**STROBE guidelines**

| **Section/topic** | **Item number** | **Recommendation** | **Reported on page** |
| --- | --- | --- | --- |
| **Title and abstract** | 1 | Indicate the study’s design with a commonly used term in the title or the abstract | 1-2 |
|  |  | Provide in the abstract an informative and balanced summary of what was done and what was found | 1-2 |
| **Introduction** |  |  |  |
| Background/rationale | 2 | Explain the scientific background and rationale for the investigation being reported | 2-6 |
| Objectives | 3 | State specific objectives, including any prespecified hypotheses | 2-3 |
| **Methods** |  |  |  |
| Study design | 4 | Present key elements of study design early in the manuscript | 2 |
| Setting | 5 | Describe the setting, locations, and relevant dates, including periods of recruitment, exposure, follow-up, and data collection | 6-8 |
| Participants | 6 | Cohort study – give the eligibility criteria, and the sources and methods of selection of participants;  describe methods of follow-up | N/A |
|  |  | Case–control study – give the eligibility criteria, and the sources and methods of case ascertainment and control selection; give the rationale for the choice of cases and controls | N/A |
|  |  | Cross-sectional study – give the eligibility criteria, and the sources and methods of selection of participants | 2-5 |
|  |  | Cohort study – for matched studies, give matching criteria and number of exposed and unexposed | N/A |
|  |  | Case–control study – for matched studies, give matching criteria and the number of controls per case | N/A |
| Variables | 7 | Clearly define all outcomes, exposures, predictors, potential confounders, and effect modifiers; give diagnostic criteria, if applicable | 3 |
| Data sources/measurement | 8* | For each variable of interest, give sources of data and details of methods of assessment (measurement); | - |
|  |  | describe comparability of assessment methods if there is more than one group | N/A |
| Bias | 9 | Describe any efforts to address potential sources of bias | - |
| Study size | 10 | Explain how the study size was arrived at | 6-8 |
| Quantitative variables | 11 | Explain how quantitative variables were handled in the analyses; if applicable, describe which groupings were chosen and why | 8-11 |
| Statistical methods | 12 | Describe all statistical methods, including those used to control for confounding | 5-6 |
|  |  | Describe any methods used to examine subgroups and interactions | 5-6 |
|  |  | Explain how missing data were addressed | N/A |
|  |  | Cohort study – if applicable, explain how loss to follow-up was addressed | N/A |
|  |  | Case–control study – if applicable, explain how matching of cases and controls was addressed | N/A |
|  |  | Cross-sectional study – if applicable, describe analytical methods taking account of sampling strategy | 6-8 |
|  |  | Describe any sensitivity analyses | - |
| **Results** |  |  |  |
| Participants | 13* | Report numbers of individuals at each stage of study – e.g., numbers potentially eligible, examined for eligibility, confirmed eligible, included in the study, completing follow-up, and analyzed | N/A |
|  |  | Give reasons for nonparticipation at each stage | N/A |
|  |  | Consider use of a flow diagram | N/A |
| Descriptive data | 14* | Give characteristics of study participants (e.g., demographic, clinical, social) and information on  exposures and potential confounders | 8-11 |
|  |  | Indicate number of participants with missing data for each variable of interest | - |
|  |  | Cohort study – summarize follow-up time (e.g., average and total amount) | N/A |
| Outcome data | 15* | Cohort study – report numbers of outcome events or summary measures over time | N/A |
|  |  | Case–control study – report numbers in each exposure category, or summary measures of exposure | N/A |
|  |  | Cross-sectional study – report numbers of outcome events or summary measures | 8-11 |
| Main results | 16 | Give unadjusted estimates and, if applicable, confounder-adjusted estimates and their precision (e.g., 95% confidence interval); make clear which confounders were adjusted for and why they were included | N/A |
|  |  | Report category boundaries when continuous variables were categorized | 11-12 |
|  |  | If relevant, consider translating estimates of relative risk into absolute risk for a meaningful time period | N/A |
| Other analyses | 17 | Report other analyses done – e.g., analyses of subgroups and interactions, and sensitivity analyses | - |
| **Discussion** |  |  |  |
| Key results | 18 | Summarize key results with reference to study objectives | 16-17 |
| Limitations | 19 | Discuss limitations of the study, taking into account sources of potential bias or imprecision; discuss both direction and magnitude of any potential bias | 19 |
| Interpretation | 20 | Give a cautious overall interpretation of results considering objectives, limitations, multiplicity of analyses, results from similar studies, and other relevant evidence | 16-17 |
| Generalizability | 21 | Discuss the generalizability (external validity) of the study results | 19 |
| **Other information** |  |  |  |
| Funding | 22 | Give the source of funding and the role of the funders for the present study and, if applicable, for the original study on which the present article is based | 1 |
